# Treatment of Gaseous Ammonia Emissions Using Date Palm Pits Based Granular Activated Carbon

**DOI:** 10.3390/ijerph17051519

**Published:** 2020-02-27

**Authors:** Muhammad Vohra

**Affiliations:** Environmental Engineering Program, Civil and Environmental Engineering Department, King Fahd University of Petroleum & Minerals (KFUPM), Dhahran 31261, Saudi Arabia; vohra@kfupm.edu.sa; Tel.: +966-013-860-2854

**Keywords:** ammonia gas, adsorption, date palm pits, activated carbon

## Abstract

The present work investigated the application of granular activated carbon (GAC) derived from date palm pits (DPP) agricultural waste for treating gaseous ammonia. Respective findings indicate increased breakthrough time (run time at which 5% of influent ammonia is exiting with the effluent gas) with a decrease in influent ammonia and increase in GAC bed depth. At a gas flow rate of 1.1 L/min and GAC column length of 8 cm, the following breakthrough trend was noted: 1295 min (2.5 ppmv) > 712 min (5 ppmv) > 532 min (7.5 ppmv). A qualitatively similar trend was also noted for the exhaustion time results (run time at which 95% of influent ammonia is exiting with the effluent gas). The Fourier Transform Infrared Spectroscopy (FTIR) findings for the produced GAC indicated some salient functional groups at the produced GAC surface including O–H, C–H, C–O, and S=O groups. Ammonia adsorption was suggested to result from its interaction with the respective surface functional groups via different mechanisms. Comparison with a commercial GAC showed the date palm pits based GAC to be having slightly higher breakthrough and exhaustion capacity.

## 1. Introduction

An exponential increase both in the human population and related industrial activities, specifically in the past century, has caused a significant increase in air pollution. Consequently several stringent environmental regulations have been promulgated, which do require an appropriate treatment of gaseous emissions, including ammonia (NH_3_), considering the respective toxicity and environmental concerns [1,2,3]. Gaseous ammonia is typically emitted from several sources including fertilizer industry [4], wastewater treatment plants [2,5], agricultural practices [6], animal feeding setups [7,8], dairy/poultry industries [9,10], composting facilities [11,12], fishmeal plants [13], gasoline vehicles [14], and from specific chemical industries [15]. Considering the respective toxicity and health concerns, various technologies have been employed for the removal of gaseous ammonia including bio-filters [3,13,16,17], catalytic systems [18] biological treatment [11], scrubbers [5] and other specific technologies such as nano-particles applications [4].

Adsorption and activated carbon based technologies have also been widely used for several environmental applications [19,20,21,22,23,24]. To that end, the conversion of agricultural wastes into granular activated carbon (GAC) and its use for pollution control applications, has also attracted much attention, considering its multi-faceted and inherent economical, and environmental advantages. Considering this, activated carbon from different carbon-rich sources including wood [1], palm shell [25], and coal and coconut shell [26] has been used for gaseous ammonia treatment. Several studies also report gaseous ammonia treatment using modified activated carbon. The respective modification methods include oxidation [27], acids [28], inorganic-agents [29], ozone [30], ZnCl_2_, HNO_3_ and (NH_4_)_2_S_2_O_8_ [26], and aluminum-zirconium poly-cations [15].

It is also important to note that extensive date fruit farming in the Middle East, North Africa, Sothern Europe, Americas (including California, Texas, Arizona, Mexico), and the Subcontinent region, produces huge agricultural wastes from the date palm trees [31]. Several studies also report green and environmental applications of date pits [32,33,34,35]. Furthermore, Jibril et al. [36] also studied production of activated carbon using date tree stem. The authors employed both acid and base activation methods. For phosphoric acid activation method, *SSA*_BET_ values reported are within 632 and 1100 m^2^/g. The highest *SSA*_BET_ value was noted at 500 °C activation temperature. For the base activation procedure (using potassium hydroxide) the *SSA*_BET_ values were within 278 and 947 m^2^/g, with the highest *SSA*_BET_ noted at 600 °C. On the other hand, Alhamed [37] reports production of activated carbon from date pits using zinc chloride chemical activation. The respective activated carbon produced at 700 °C showed *SSA*_BET_ value of 951 m^2^/g. Also, Al-Muhtaseb et al. [38] report production of activated carbon from date pits with *SSA*_BET_ value of 690 m^2^/g using physical activation at 900 °C. Belhachemi et al. [39] also employed date pits with physical activation for the production of activated carbon at 800 °C. The authors prepared several activated carbon samples under a varying set of conditions and report *SSA*_BET_ values between 652 and 1669 m^2^/g. Bouchelta et al. [40] also report production of activated carbon from date pits with *SSA*_BET_ value up to 635 m^2^/g using physical activation. Daifullah and Girgis [41] report production of activated carbon from date pits using chemical activation. The respective activated carbon showed *SSA*_BET_ value of 771 m^2^/g. El-Naas et al. [42] also report production of activated carbon from date pits using carbon dioxide at 900 °C with *SSA*_BET_ value 490 m^2^/g. Girgis and El-Hendawy [43] also report production of activated carbon from date pits using phosphoric acid based activation under a varying set of conditions. Results showed *SSA*_BET_ values up to 945 m^2^/g at 700 °C. Hameed et al. [44] also report production of activated carbon from date pits employing potassium hydroxide and report *SSA*_BET_ value 763 m^2^/g at 850 °C. Merzougui and Addoun [45] also report production of activated carbon from date pits using chemical activation under a varying conditions and indicate *SSA*_BET_ values up to 1040 m^2^/g.

The above given literature review shows the production of GAC from date pits, which has also been successfully used for several water treatment applications. Furthermore, the aforementioned literature review also indicated the importance of toxic gaseous ammonia removal from the respective contaminated streams. However, the application of date palm pits based GAC for the treatment of gaseous ammonia pollution control, has not been explored. Nevertheless, environmental regulations do require appropriate treatment of toxic gaseous emissions including ammonia (NH_3_) considering the respective health concerns. The present work therefore focuses on gaseous ammonia treatment using GAC derived from date palm pits. Effect of process variables including the influent gas flow rate, gas concentration, and GAC column length (bed depth) on to ammonia gas adsorption was investigated. Results as reported in this communication show that DPP based GAC can successfully treat gaseous ammonia under a varying set of process conditions.

## 2. Materials and Methods

### 2.1. Materials

Some specific chemicals and materials that were used in the present work included the following: pH calibration standards (FISHER), phosphoric acid (KH_2_PO_4_ 85% *w*/*w*; BAKER), Liquid Nitrogen (at 77 K), and ammonia standard gas (100 ppmv). The high purity air was generated using a zero air setup (Thermo Scientific, Model 111, Waltham, MA, USA). A commercial GAC sample, i.e., Filtrasorb 400 (Calgon, Moon Township, PA, USA), was also employed for gaseous ammonia adsorption and the outcomes were compared with the respective date pits based GAC results to realize latter’s efficiency.

### 2.2. Granular Activated Carbon (GAC) Production

Initially the date palm pits were acquired from a local source, and were then reduced to size of 1–1.2 mm using a professional cutter. The respective date pits particles were then converted into GAC using a chemical activation process [46]. For the initial impregnation step, the date pits particles were first dried in an oven at 100 °C. The respective date particles and phosphoric acid (60% *w*/*w*) were mixed at impregnation ratio of 1.6 mL/g (acid/date-pits). This mixture was left overnight for soaking. Then the respective mixture was shifted to 25 mm × 300 mm (diameter × length) stainless steel tubes, which had exit holes at both ends (to allow the produced gases and vapors to exit). These stainless steel setup was later on shifted to a professional furnace (Lenton, Hope Valley, UK). After this, the furnace temperature was raised to 500 °C, at a gradual increase rate of 10 °C per minute. The respective tubes holding the acid/date-pits mixture, remained in the furnace for 2 h. After this step, the furnace temperature was gradually cooled to the room temperature, after which the tubes were transferred to a standard desiccator. The end product as retrieved from the tubes was continuously washed using high purity water (CORNING Mega Pure^TM^ System). This process of washing continued till the washing water pH reached to near neutral. The resulting product was dried at 110 °C using a standard oven (Fisher Scientific, Hampton, NH, USA), and then appropriately sieved to obtain around 1 mm particles employing U.S. sieve size # 18.

### 2.3. Gaseous Ammonia Dynamic Adsorption Studies

**Figure 1:**
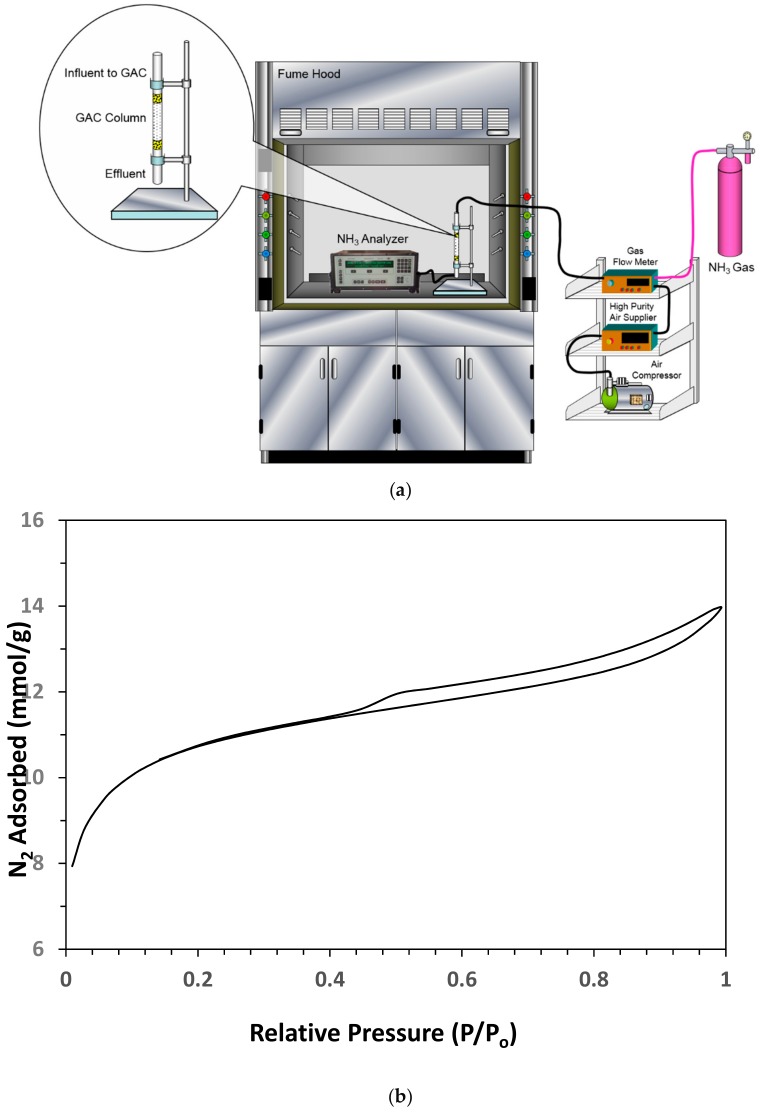

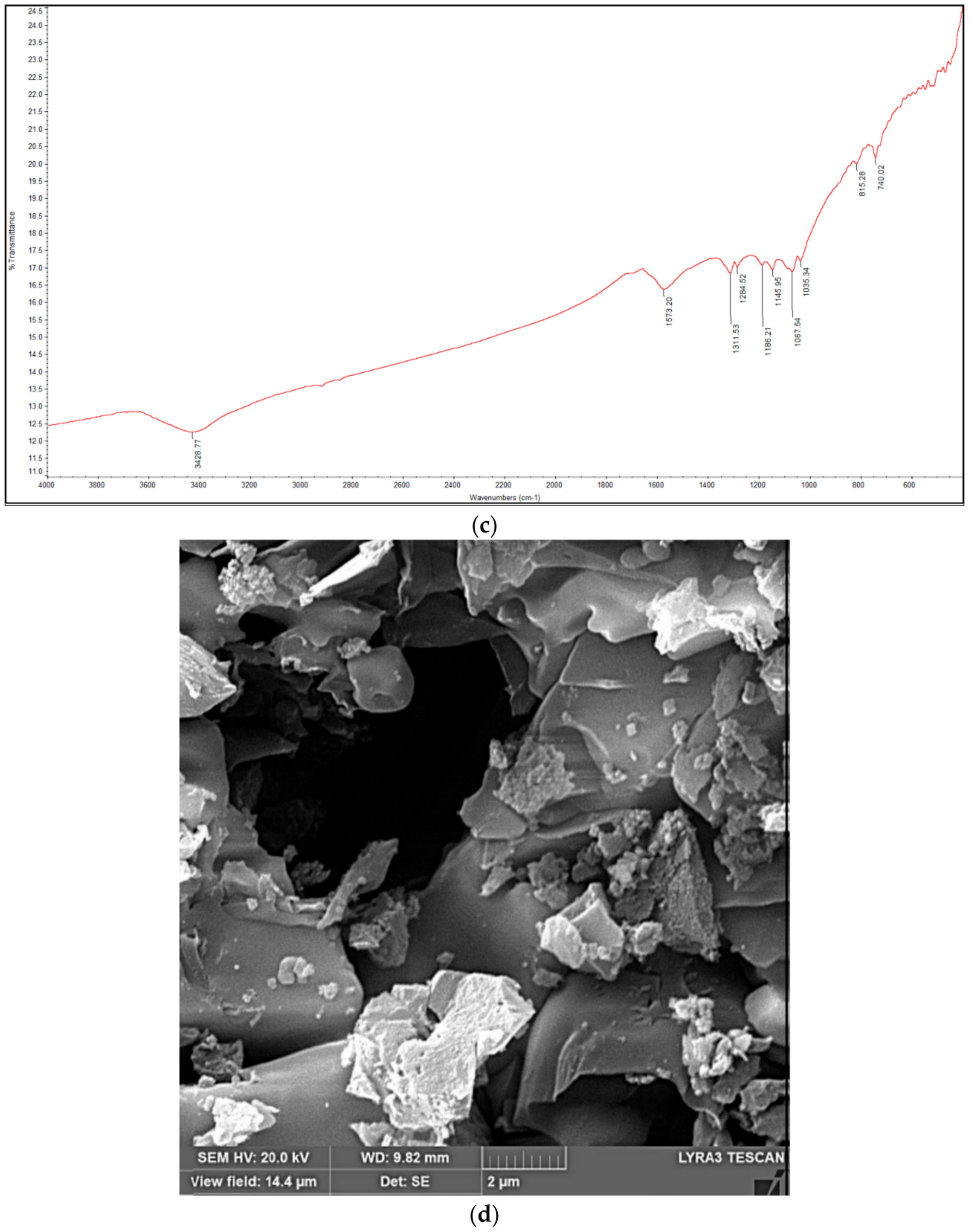
(**a**) The experimental setup used for the dynamic continuous ammonia gas flow adsorption experiments. (**b**) The BET adsorption isotherm findings for the date pits granular activated carbon (GAC) (Preparation conditions: Phosphoric acid (60% *w*/*w*); Impregnation ratio 1.6 mL/g (acid/date-pits); Furnace temperature 500 °C for 2 h). (**c**) The transmittance vs wavenumber Fourier Transform Infrared Spectroscopy (FTIR) findings for the produced GAC (Preparation conditions: Phosphoric acid (60% *w*/*w*); Impregnation ratio 1.6 mL/g (acid/date-pits); Furnace temperature 500 °C for 2 h). (**d**) SEM findings for the GAC produced from date palm pits (Preparation conditions: Phosphoric acid (60% *w*/*w*); Impregnation ratio 1.6 mL/g (acid/date-pits); Furnace temperature 500 °C for 2 h).

The dynamic adsorption studies were completed using a bench scale experimental setup [47]. Figure 1a provides the layout of respective setup that was used for the gaseous ammonia dynamic adsorption experiments. It consisted of following major individual components: Ammonia gas standard cylinder, high purity air supply unit, standard flow meters connecting the gas sources to the activated carbon column via a series of valves to control gas flow and gas concentration, GAC column, and ammonia gas analyzer. The column containing the produced GAC (through which gaseous ammonia was passed continuously) was made of Fluorinated Ethylene Propylene (FEP) tubing with an inner diameter of 6.35 mm and outer diameter of 7.938 mm. The column was first provided with an inert support at the bottom followed by GAC filling that was then topped with the same inert material. The specific and separate gas flows from the high purity air supply source (Thermo Scientific, Model 111, Waltham, MA, USA) and the standard ammonia gas cylinder were obtained using separate/dedicated standard flow controllers (Cole Palmer, Vernon Hills, IL, USA). After this, the continuous streams from the high purity air source and ammonia gas standard were mixed (at specific flow rates) to realize the desired influent gaseous ammonia concentration (for the adsorption experiment). The respective gaseous ammonia stream was then continuously passed in a down flow direction through the above mentioned GAC column. The effluent/exiting gas (after treatment) was then tested as described in Section 2.4. The gaseous ammonia removal experiments were conducted at different process conditions including varying flow rates and ammonia concentrations. The obtained responses included the Breakthrough Time (run time at which 5% of influent ammonia is exiting the column) and Exhaustion Time (run time at which 95% of influent ammonia is exiting the column). To obtain these responses, the effluent ammonia results were plotted against time for the whole test duration.

### 2.4. Analytical Methods

The specific surface area (*SSA_BET_*) of the produced activated carbon was determined employing a state-of-the-art physio-sorption setup (ASAP 2020, Micromeritics, Norcross, GA, USA). The conventional nitrogen adsorption equilibrium data as given in Figure 1b was eventually employed for the respective analysis. The specific surface area (*SSA_BET_*) value of produced GAC was determined to be 822 m^2^/g. The respective average pore width was determined to be 23.01 Å ((4V/A) by BET)). The results indicated most specific surface area, i.e., 735 m^2^/g to be in the porous region of produced GAC. The total carbon content (*w*/*w*) of respective activated carbon was determined to be around 82% (AnalytikJena Multi EX 2000, Jena, Germany). The Fourier Transform Infrared Spectroscopy (FTIR) characterization of the produced date pits based GAC was also completed employing the Fourier transform infrared technique (FTIR, Thermo Scientific, Waltham, MA, USA). The respective findings are given in Figure 1c. The salient functional groups, as noted from the respective FTIR characterization, at the GAC surface, included O-H, C-H, C–O, and S=O. The respective surface functional groups are expected to initiate ammonia uptake by the activated carbon. Furthermore, hydrophobic interactions and hydrogen bonding transpiring between the ammonia molecules and surface oxygen based functional groups can also initiate and enhance gaseous ammonia adsorption on activated carbon surface [22]. These results are invoked later to explain the ammonia adsorption results. The effluent/exiting gas (after treatment) from dynamic continuous gas flow experiments was tested using a state-of-the-art gaseous ammonia analyzer (Model 17i, Thermo Scientific, Waltham, MA, USA). Furthermore, Figure 1d provides the SEM results for the date pits based GAC. The respective results indicate a porous structure that eventually helps to adsorb the target pollutants that is similar to as noted in previous studies for activated carbon [48].

## 3. Results and Discussion

### 3.1. Effect of Ammonia Gas Concentration

**Figure 2:**
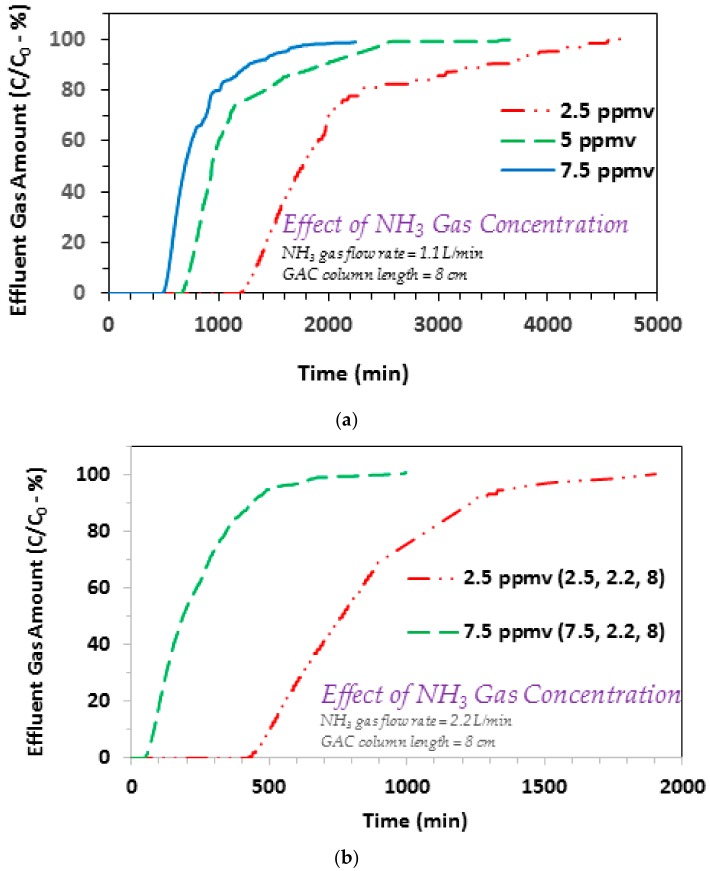
(**a**) Effect of influent ammonia gas concentration on the adsorption breakthrough curve profile of ammonia gas on to GAC produced from date palm pits. (**a**) GAC *SSA_BET_* = 822 m^2^/g and average pore width = 23.01 Å; ammonia/NH_3_ gas flow rate = 1.1 L/min; influent ammonia/NH_3_ gas concentrations 2.5, 5, and 7.5 ppmv; GAC column length = 8 cm; GAC bed dia. 6.35 mm. (**b**) GAC *SSA_BET_* = 822 m^2^/g and average pore width = 23.01 Å; ammonia/NH_3_ gas flow rate = 2.2 L/min; influent ammonia/NH_3_ gas concentrations 2.5, and 7.5 ppmv; GAC column length = 8 cm; GAC bed dia. 6.35 mm.

**Figure 3:**
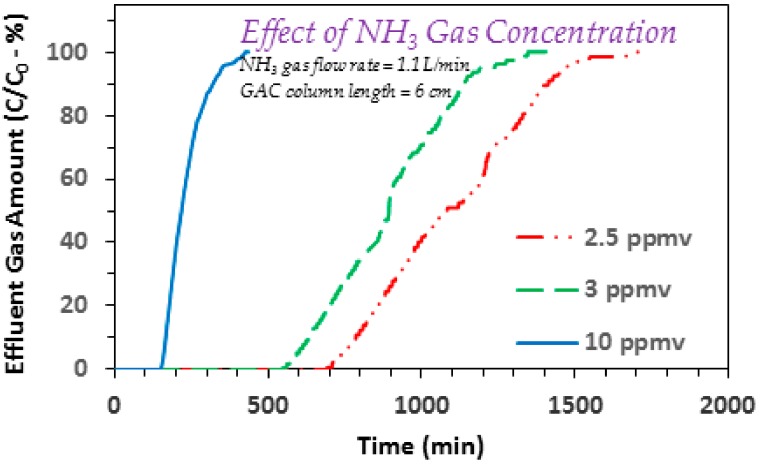
Effect of influent ammonia gas concentration on the adsorption breakthrough curve profile of ammonia gas on to GAC produced from date palm pits (GAC *SSA_BET_* = 822 m^2^/g and average pore width = 23.01 Å; ammonia/NH_3_ gas flow rate = 1.1 L/min; influent ammonia/NH_3_ gas concentrations 2.5, 3, and 10 ppmv; GAC column length = 6 cm; GAC bed dia. 6.35 mm).

Initially, a set of experiments were conducted to assess the effect of influent ammonia concentration on to its adsorption based removal. To that end, several experiments were conducted at varying influent gaseous ammonia concentrations, using GAC column lengths of 6 and 8 cm. The initial experiments were conducted between 2.5 and 7.5 ppmv gaseous ammonia for the 8 cm GAC column. The respective findings are shown in Figure 2a,b, at gas flow rates of 1.1 and 2.2 L/min, respectively. It is evident that with a gradual decrease in the influent ammonia concentration, the breakthrough point also shifts towards longer times. For example, the results in Figure 2a show the following breakthrough trend: 1295 min (2.5 ppmv) > 712 min (5 ppmv) > 532 min (7.5 ppmv). Furthermore, the exhaustion time values for the respective experiments also show higher run times at lower gaseous ammonia concentrations, i.e., 4000 min (2.5 ppmv) > 2312 min (5 ppmv) > 1574 min (7.5 ppmv). Furthermore, the adsorption trends in Figure 2 typically show a broader adsorption breakthrough curve that indicates comparatively larger length of the mass transfer zone (MTZ). This is somewhat different from the adsorption of benzene onto DPP based GAC that showed a sharp breakthrough curve [24]. Such a different trend in case of ammonia may be ascribed to basic nature of ammonia moieties that can interact with the surface acidic groups [29]. An increase in the incoming gaseous ammonia and its transfer from the bulk gas phase to the surface of GAC, will initiate a faster consumption of GAC surface adsorption sites (including those in the pores). As the respective surface adsorption sites are fixed and limited, it is expected that an earlier breakthrough and also an earlier exhaustion, will transpire at higher influent ammonia concentrations. Furthermore, additional experiments conducted under similar conditions but at 2.2 L/min, also show qualitatively similar trends (Figure 2b). The effect of varying influent gaseous ammonia concentration on to breakthrough/exhaustion was further investigated using a 6 cm GAC column at 3 and 10 ppmv ammonia. The respective findings are given in Figure 3. Similar to Figure 2 findings, an increase in the gas concentration from 3 to 10 ppmv, reduces the breakthrough time from 595 and 159 min, and the exhaustion time from 1231 to 354 min.

Further comparing the results for 2.5 and 5 ppmv systems from Figure 2a, the decrease in breakthrough and exhaustion time values is approximately proportional to the respective increase in ammonia concentration. This shows that differences between the mass of ammonia transferred from the bulk gas phase to the GAC surface at both flow rates, is minimal. This suggests that the bulk diffusion and pore diffusion of gaseous ammonia is sufficiently high and therefor the overall mass transfer is not significantly affected by the respective increase in bulk ammonia gas concentration. A similar trend is also noted comparing the respective breakthrough time values (595 and 159 min) and the exhaustion time values (1231 and 354 min), for the 3 and 10 ppmv ammonia systems using 6 cm GAC column (Figure 3). Considering the importance of this point, especially from practical applications point of view, one additional experiment was conducted at 2.5 ppmv and the breakthrough and exhaustion time values were noted to be 747 and 1476 min, respectively (Figure 3). Comparing these results with the 10 ppmv findings (Figure 3) that showed breakthrough and exhaustion time values of 159 and 354 min, respectively, an increase in influent ammonia gas concentration to four times causes an approximately similar decrease in both the breakthrough and exhaustion times. This observation was further explored by investigating the effect of varying gaseous ammonia flow rates and GAC column lengths, on the respective breakthrough/exhaustion time trends, with details provided in the sections given below.

### 3.2. Effect of Ammonia Gas Flow Rate

**Figure 4:**
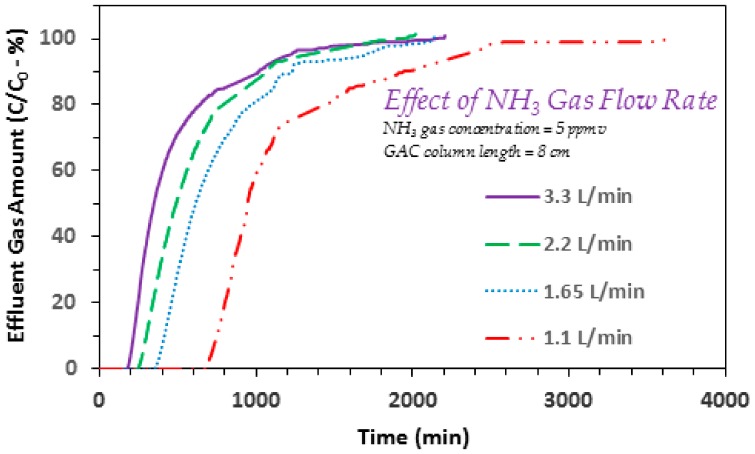
Effect of influent ammonia/NH_3_ gas flow rate on the adsorption breakthrough curve profile of ammonia gas on to GAC produced from date palm pits (GAC *SSA_BET_* = 822 m^2^/g and average pore width = 23.01 Å; ammonia/NH_3_ gas flow rates = 1.1, 1.65, 2.2, and 3.3 L/min; influent ammonia/NH_3_ gas concentrations 5 ppmv; GAC column length = 8 cm; GAC bed dia. 6.35 mm).

The present study also investigated the effect of gas flow rate on to ammonia adsorption using the date pits based GAC. Figure 4 provides the respective results for gas flow rates between 1.1 to 3.3 L/min (at an influent gaseous ammonia concentration of 5 ppmv and GAC column length of 8 cm). Typically, a gradual increase in the ammonia gas flow rate also results in a gradual decrease in the respective breakthrough time and exhaustion time responses (Figure 4). For example, the breakthrough times at flow rates of 1.1, 1.65, 2.2, and 3.3 L/min are noted to be 712, 383, 272, and 197 min, respectively (Figure 4). Hence higher breakthrough time values transpire at lower ammonia gas flow rates. Furthermore, exhaustion times of 2312, 1673, 1315, and 1213 min are also noted for 1.1, 1.65, 2.2, and 3.3 L/min flow rate studies, respectively. This is similar to the aforementioned breakthrough time trend that can be explained as follows. The mass transfer rate of ammonia molecules from gas phase to GAC surface is also comparatively small at lower influent gas flow rates. Hence the GAC surface sites being occupied per unit time is also lesser, yielding higher breakthrough time and exhaustion time responses as compared to those at higher gas flow rates (Figure 4). These findings are important especially for practical applications. To build on these important results, the effect of GAC column length on gaseous ammonia removal was also studied, as given below.

### 3.3. Effect of GAC Column Length

**Figure 5:**
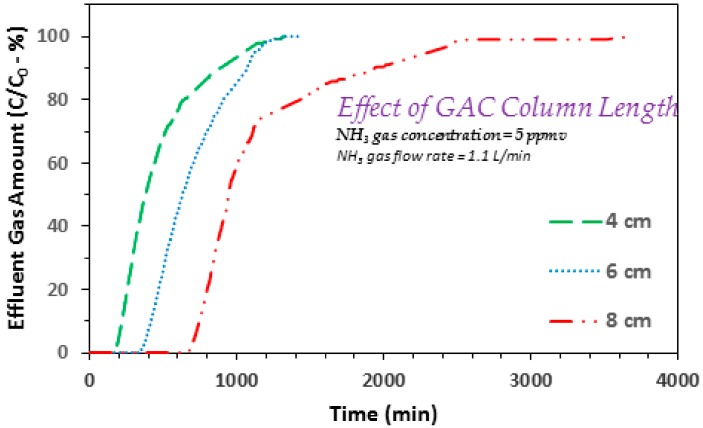
Effect of GAC column length on the adsorption breakthrough curve profile of ammonia gas on to GAC produced from date palm pits (GAC *SSA_BET_* = 822 m^2^/g and average pore width = 23.01 Å; ammonia/NH_3_ gas flow rate = 1.1 L/min; influent ammonia/NH_3_ gas concentrations 5 ppmv; GAC column lengths = 4, 6, and 8 cm; GAC bed dia. 6.35 mm).

Another set of experiments conducted at varying GAC column lengths (and 1.1 L/min and 5 ppmv gas flow rate and concentration, respectively) indicated higher breakthrough and exhaustion time values for larger GAC column depths (Figure 5). The 4, 6, and 8 cm GAC column lengths show breakthrough at 197, 385, and 712 min, respectively, whereas the respective exhaustion time values are 1054, 1134, and 2312 min. Both trends show an increase as the GAC column length increases from 4 to 8 cm. To further assess this subject, another experiment was conducted using the shortest (4 cm) GAC column at three times higher flow rate of 3.3 L/min (and 5 ppmv). This caused a reduction in the breakthrough time from 197 min (for 1.1 L/min) to 51 min (for 3.3 L/min). An earlier study also indicated higher gaseous ammonia adsorption with an increase in the initial (zinc chloride impregnated) activated carbon sample [49]. However, above an optimum zinc chloride amount, the adsorption capacity actually decreased. The authors ascribed such a decrease in gaseous ammonia adsorption to the agglomeration of zinc chloride moieties on to activated carbon surface. Furthermore, and as also stated earlier that the adsorption trends in Figure 2, Figure 3, Figure 4 and Figure 5, typically show a broader adsorption breakthrough curve that indicates comparatively larger length of the mass transfer zone (MTZ). This is different from the adsorption of benzene onto DPP based GAC that showed a sharp breakthrough curve [24]. Such a different trend in case of ammonia may be ascribed to the *basic* nature of ammonia moieties that can interact with the surface acidic groups [29]. Furthermore, the adsorbed ammonia can also dissolve in moisture within the pore space, thus resulting into its accumulation and buildup. This will cause formation of cationic ammonium species that could possibly interact with the surface of activated carbon via surface functional groups, acid–base reactions, and also through van der Waals interactions, which are simplified as given below [29].

**N**H_3_ (gas) ↔ **N**H_3_ (aqueous) (1)

**N**H_3_ (aqueous) + H^+^ ↔ **N**H_4_^+^ (aqueous) (2)

**GAC**-O^−^ + **N**H_4_^+^ ↔ **GAC**-O-**N**H_4_ (3)

These mechanisms could also possibly explain the broader ammonia adsorption curves a noted in Figure 2, Figure 3, Figure 4 and Figure 5. Furthermore, to check out the data reproducibility, two experiments for 5 ppmv ammonia gas concentration, at 1.1 L/min gas flow rate and GAC column length of 4 cm, yielded breakthrough time values of 197 and 227 min. Similarly, another set of experiments completed at 5 ppmv ammonia gas concentration, 1.1 L/min gas flow rate and GAC column length of 8 cm, yielded breakthrough time values of 712 and 750 min. These values are close, especially for the bench scale dynamic flow studies. It should also be noted that real life applications require the GAC column length to be larger than the critical GAC bed depth, i.e., column length just enough to avoid breakthrough at time zero. These observations suggest a longer GAC column length for gaseous ammonia adsorption. These findings show that with a careful selection of respective operational design parameters, the date palm pits based GAC can be successfully used for gaseous ammonia treatment. To build on these encouraging results, a commercial GAC was also used for comparison purpose, and the respective findings are given below.

### 3.4. Comparison with a Commercial GAC

**Figure 6:**
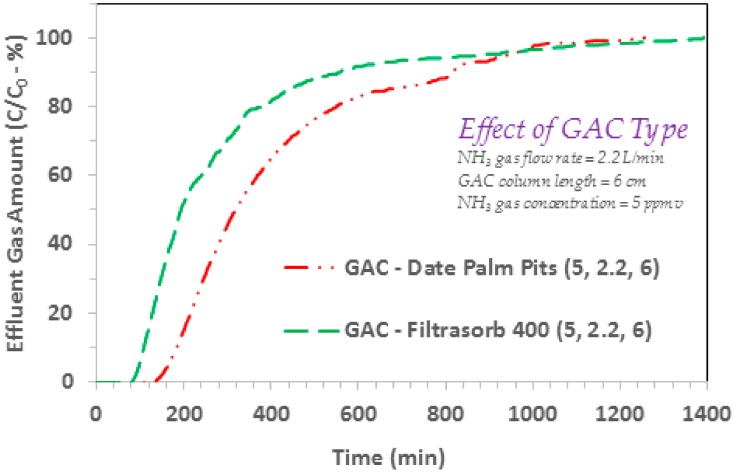
Effect of GAC type on the adsorption breakthrough curve profile of ammonia gas on to GAC produced from date palm pits (*SSA_BET_* 822 m^2^/g for date palm pits based GAC and 1100 m^2^/g for Filtrasorb 400 GAC; ammonia/NH_3_ gas flow rate = 2.2 L/min; influent ammonia/NH_3_ gas concentrations 5 ppmv; GAC column length = 6 cm; GAC bed dia. 6.35 mm).

The efficiency of GAC produced from date palm pits was also compared with a commercial GAC, i.e., Filtrasorb 400 (Calgon, Moon Township, PA, USA). The respective findings (at ammonia gas flow rate 2.2 L/min; influent ammonia gas concentration 5 ppmv; GAC column length 6 cm) as given in Figure 6, show higher breakthrough and exhaustion capacity for the date palm pits based GAC. The average pore width of the produced GAC, which is ~23 Å, is much wider than 3.26 Å molecular-size of ammonia [2,50]. This makes it very conducive for the ammonia molecules to diffuse through the pores into the pore-volume of produced GAC, which also holds majority of specific surface area, i.e., 735 m^2^/g. Furthermore, the aforementioned discussion on higher bulk and pore diffusion of gaseous ammonia, yielding larger gaseous ammonia mass transfer from the bulk gas phase to the produced GAC surface at higher ammonia concentrations (Figure 2 and Figure 3), can also explain the noted better gaseous ammonia efficiency of produced GAC as compared to the commercial GAC (Figure 6). It has also been reported that gaseous ammonia moieties can initiate hydrogen bonding with the acidic functional groups at the GAC surface (in an acid–base interaction), with higher adsorption noted for the activated carbons with larger surface acidic groups [30,51,52]. In one study, which observed reduced specific surface area of GAC with ozone treatment, still an increased introduction of surface acidic groups (because of ozone treatment) yielded higher ammonia removal efficiency [30]. In another study, an increased gaseous ammonia uptake for activated carbon modified with different acids was also noted to be proportional to the introduction of acidic surface functional groups [28], and also oxygen surface functional groups [25,26]. In the present investigation, employing the FTIR findings indicated some salient functional groups at the produced GAC surface including O-H, C-H, C–O, and S=O groups. It is also interesting to note that the gaseous ammonia species because of its molecular structure and electrons distribution, can serve both as a hydrogen-bond donor and acceptor [51]. Hence the gaseous ammonia molecule is in a unique position to bond with the above mentioned functional groups at the produced GAC surface. Wu et al. [22] who studied adsorption of N-based atrazine pollutant onto several activated carbon samples, both in the absence and presence of Bisphenol A, discuss several possible atrazine uptake mechanisms. It was noted that factors including activated carbon’s polarity, specific surface area, and porosity, did affect atrazine adsorption on to activated carbon. Also hydrophobic interactions, hydrogen bonding, and π–π exchanges, were considered to initiate adsorption. For example, hydrogen bonding between the N-H moieties and surface carbonyl groups, was indicated to cause atrazine uptake by the activated carbon surface. Similarly, hydrophobic interactions and hydrogen bonding transpiring between the ammonia molecules and surface oxygen based functional groups, can also initiate and enhance gaseous ammonia adsorption on to activated carbon surface. Additionally, other forces including the van der Waals interactions, can also contribute towards ammonia adsorption. In summary, the findings reported in the present work are promising, paving the path for practical applications of date palm pits based GAC for gaseous ammonia treatment.

## 4. Conclusions

The application of GAC derived from date palm pits (DPP) agricultural waste for treating gaseous ammonia was studied along with the effect of influent gas flow rate, gas concentration, and GAC column length (bed depth) on to ammonia gas adsorption. Results show that DPP based GAC successfully treats gaseous ammonia under a varying set of process conditions. Respective findings indicate increased breakthrough/exhaustion time with a decrease in influent ammonia and increase in GAC bed depth. The broader breakthrough curves as noted in the present case for gaseous ammonia adsorption indicate comparatively larger length of mass transfer zone (MTZ), which suggests that longer GAC column lengths should be provided to avoid early breakthrough, for optimum real life applications. The efficiency of GAC produced from date palm pits was also compared with a commercial GAC and it showed the date palm pits based GAC to be having slightly higher breakthrough and exhaustion capacity. In conclusion, results from the present work show that the DPP based GAC can be successfully used to treat gaseous ammonia emissions with potential applications in many industries.
